# Item analysis on the quality of life scale for anxiety disorders QLICD-AD(V2.0) based on classical test theory and item response theory

**DOI:** 10.1186/s12991-024-00504-2

**Published:** 2024-05-10

**Authors:** Hongqiang Shi, Yu Ren, Junding Xian, Haifeng Ding, Yuxi Liu, Chonghua Wan

**Affiliations:** 1 The First Dongguan Affiliated Hospital of Guangdong Medical University, Dongguan, China; 2 School of Humanities and Management, Key Laboratory for Quality of Life and Psychological assessment and Intervention, Guangdong Medical University, Dongguan, China; 3 Affiliated hospital of Guangdong medical university, Zhanjiang, 524000 China

**Keywords:** Quality of life, Anxiety disorder, Classical test theory, Item response theory

## Abstract

**Background:**

Anxiety disorders can cause serious physical and psychological damage, so many anxiety scales have been developed internationally to measure anxiety disorders, but due to the cultural differences and cultural dependence of quality of life between Chinese and Western cultures, it is difficult to reflect the main characteristics of Chinese patients. Therefore, we developed a scale suitable for Chinese patients with anxiety disorders: the Anxiety Disorders Scale of the Quality of Life Instruments for Chronic Diseases (QLICD-AD), hoping to achieve satisfactory QOL assessments for anxiety disorders.

**Objectives:**

Items from the Anxiety Disorders Scale of the Quality of Life in Chronic Disease Instrument QLICD-AD system were analyzed using CTT and IRT to lay the groundwork for further refinement of the scale to accurately measure anxiety disorders.

**Methods:**

120 patients with anxiety disorder were assessed using the QLICD-AD (V2.0). Descriptive statistics, variability method, correlation coefficient method, factor analysis and Cronbach’s coefficient of CTT, and graded response model (GRM) of item response theory were used to analyze the items of the scale.

**Result:**

CTT analysis showed that the standard deviation of each item was between 0.928 and 1.466; Pearson correlation coefficients of item-to-domain were generally greater than 0.5 and also greater than that of item-to-other domain; the Cronbach ‘s of the total scale was 0.931, α of each domain was between 0.706 and 0.865. IRT analysis showed that the discrimination was between 1.14 and 1.44. The difficulty parameter of all items increased with the increase of grade. But some items (GPH6,GPH8,GPS3,GSO2-GSO4,AD2,AD5) difficulty parameters were less than 4 or greater than 4. The average of information amount was between 0.022 and 0.910.

**Conclusion:**

Based on CTT and IRT analysis, most items of the QLICD-AD (V2.0) scale have good performance and good differentiation, but a few items still need further revision. Suggests that the QLICD-AD (V2.0) appears to be a valid measure of anxiety disorders. It may effectively improve the diagnosticity of anxiety disorders, but due to the limitations of the current sample, further validation is needed in a broader population extrapolation trial.

## Background

Anxiety disorders (AD) have become the most common type of mental disorder in the population, often leading to chronic illness and disability [1]. Anxiety disorders are characterized by excessive and persistent fear, anxiety, or avoidance of perceived threats, and may include panic attacks [2]. The social pressure of China’s adults is increasing along with the high development of China’s society and economy. According to a research held by Huang, a China Mental Health Survey in 2012 showed that anxiety disorders were the most common class of disorders both in the 12 months before the interview (weighted prevalence 5.0%, 4.2–5.8) and in lifetime (7.6%, 6.3–8.8) [3]. Impact on the mental health of the community population during the COVID-19 pandemic, primarily in terms of depressive and anxiety symptoms [4]. At the same time, an American study showed that anxiety disorders have the highest estimated lifetime prevalence rates of any psychiatric disorder. (18.0–3.7%) [5]. A survey found that the prevalence of anxiety symptoms among Chinese older adults (≥ 60 years) was 12.15% (1751/14,417), and the prevalence of anxiety disorders among older adults had nearly tripled in six years [6]. The average annual family medical cost of mental illness has increased from $1094.8 to $3665.4 [7], resulting in a strain on health care resources and an increase in the socioeconomic burden on families. In addition, due to the lack of assessment criteria, many people classify anxiety disorders as depression, which leads to later worsening of the illness and makes differential diagnosis increasingly difficult [8].

Although the Generalized Anxiety Scale (GAD-7) has been used in clinical practice in China, we found that it focuses only on psychological aspects and does not include physical conditions and social support, which is not well suited to the Chinese context. Meanwhile, in China, SF-36 and WHOQOL-BREF are mostly used to measure the QOL of anxiety disorder patients, but we think they lack pertinence. Some scholars believe that QOL should use a combination of generic and specific instruments to maximize both sensitivity and generalizability [9].

Although it is possible to develop Chinese versions of Western scales after a rigorous translation process, their Chinese versions are hardly responsive to Chinese characteristics due to the differences between Western and Chinese cultures and the strong cultural dependence of quality of life. Considering the culture dependence and disease pertinence of QOL, we systematically developed a QOL instrument system called QLICD(V2.0) (Quality of Life Instruments for Chronic Diseases) [10–12]. Among them, QLICD-AD (V2.0) is a specific scale for anxiety disorders, which is composed of the 28-item general module and the 12-item specific module. The results of preliminary validation showed that it has good psychometric properties [13–14].

The quality of the items is an important aspect of the quality of the scale. Item analysis is an integral part of the scale development, application and simplification. The classical test theory (CTT) is a tool for evaluating assessments from a macro perspective, with low sample size requirements for simplicity and conceptual intuition for parameter estimation of the model. However, the development of the whole scale system is still mainly based on the CTT, and there are some obvious shortcomings, such as the sample dependence of the statistics, the ambiguity of the error and imprecision of the reliability estimation, and the inconsistency between the ability and difficulty scales [15]. While item response theory (IRT) is widely used in micro aspects, such as item analysis in psychological and educational measurements, with the advantages of sample freedom and accuracy of results, to further deepen the analysis of the quality of the scale, providing more detailed and detailed and standardized [16–17], while the IRT is more computationally intensive, and the results of the analysis of small samples may be unstable [18]. Combining the two methods to analyze the entries can compensate for their respective shortcomings and greatly improve the level and scientificity of scale development and evaluation. Therefore, in our study, CTT and IRT were used to analyze the items together from both macro and micro aspects, thus avoiding the errors caused by relying only on statistical analysis and improving the representativeness and reliability of the items.

The purpose of this study was to systematically evaluate the items of the QLICD-AD(V2.0) based on classical test theory and item response theory, which will provide a basis and reference for further optimization and application of the scale. Also it will help to evaluate applicability of the QLICD-AD (V2.0) to hospitalized patients with anxiety disorders that effectively facilitates the assessment of quality of life in patients with AD.

## Methods

### Participants

We recruited participants at the Affiliated Hospital of Guangdong Medical University in China using following inclusion and exclusion criteria. The diagnosis was fully supported by the Department of Psychiatry at the affiliated Hospital of Guangdong Medical University.

Inclusion Criteria: ①Participants should meet the diagnostic criteria of ICD-10 (International Classification of Diseases).②Participants should have clear consciousness and stable condition. ③Participants should be able to complete the questionnaire on their own. ④Participants should be willing to participate in this research and have signed an informed consent form.

Exclusion Criteria:① Participants with anxiety disorders caused by organic and somatic brain diseases.②Participants who were diagnosed by the use of psychoactive substances or have a history of using psychoactive substances. ③Participants who are delirious and in the acute phase of an anxiety disorder. Participants who have been diagnosed with any other mental illness.

After explaining the study procedure to eligible patients, we sign an informed consent form with them. The study protocol and informed consent form were approved by the Institutional Review Board (IRB) of the investigator’s institution of the investigator’s institution.

### Measurement tools

QLICD-AD(V2.0): The second edition of Quality of Life Instruments for Chronic Diseases-Anxiety Disorder (QLICD-AD, V2.0) are combined with general module and anxiety disorder module, 40 items in total [14, 19]. General module includes 3 domains which are physical function (GPH1-GPH9), social function (GSO1-GSO8) and psychological function (GPS1-GPS11), and 9 facets, 28 items in total. Anxiety disorder module includes 12 items. Each item is a five-level item (possible score range: 1 to 5, ranging from 1 no problem to 5 extreme problem). According to score principle, it can calculate the standard score of each domain, facet and the total. The standard score of it is from 0 to 100, the more score, the higher QOL. Details of the items were presented in Table 1.

**Table 1:** Items of the QLICD-AD (V2.0)

Item	Item description	Item	Item description	
1. GPH1	Have you had a good appetite?	21. GSO1	Could you socialize with others like before the illness?	
2. GPH2	Were you satisfied with your sleep?	22. GSO2	Have you had good relations with your families?	
3. GPH3	Has the disease or treatment affected your sexual activities?	23. GSO3	Have you had good relations with your friend?	
4. GPH4	Have you had normal bowel movements?	24. GSO4	Could you acquire material and emotional help and support from your family when you need?	
5. GPH5	Have you felt pain or uncomfortable?	25. GSO5	Could you get the care and support from your friends and relatives?	
6. GPH6	Could you take care of your daily life?	26. GSO6	Has the economic problems caused by illness or treatment affected your life?	
7. GPH7	Could you work?	27. GSO7	Has the disease or treatments interfered with your work or housework?	
8. GPH8	Could you walk independently?	28. GSO8	Could you assume the appropriate family role?	
9. GPH9	Have you felt fatigue easily?	29. AD1	Have you ever been scared for no reason?	
10. GPS1	Could you do something with concentration?	30. AD2	Have you Frequent or urgent urination?	
11. GPS2	Have your memory and concentration been affected by the disease?	31. AD3	Have you Feeling of dying or madness?	
12. GPS3	Have you found fun in life?	32. AD4	Have you consider yourself seriously ill?	
13. GPS4	Have you felt fretful or irritable?	33. AD5	Were you tired or drowsy after taking this medicine?	
14. GPS5	Have you thought yourself as the burden of the family?	34. AD6	Have you been troubled or restless?	
15. GPS6	Have you been worried about your disease?	35. AD7	Have you experienced chest tightness, palpitations, or choking?	
16. GPS7	Have you felt depressed or sad?	36. AD8	Have you often felt tingling and trembling in your hands and feet?	
17. GPS8	Have you felt pessimism and despair?	37. AD9	Have you had abdominal discomfort?	
18. GPS9	Have you been afraid of your illness?	38. AD10	Have you had trouble sleeping because of daydreaming?	
19. GPS10	Were you optimistic about your disease?	39. AD11	Were you afraid of certain places or something?	
20. GPS11	Have you worsened your temper because of illness?	40. AD12	Were you worried about others knowing about your illness?	

### Statistical analysis

After collecting the data from the completed scale, the demographic profile was first described after data organization. Then the statistical indicators in the CTT were calculated separately as well as derived using the graded response model (GRM) to calculate the average amount of information, coefficient of difficulty, and discrimination in the IRT. All the above analyses were performed in R studio.

### Classical test theory(CTT)

CTT is founded on the proposition that measurement error, a random latent variable, is component of the observed score random variable [19, 20]. It is a traditional quantitative approach to testing the reliability and validity of a scale based on its items [21].

The CTT was analyzed for reliability and validity, and the scale items were evaluated in this study using four statistical methods: the Cronbach’s coefficient method, the variability method, the correlation coefficient method, and the factor analysis method. The items that satisfy at least three of these statistical methods can be comprehensively evaluated as good items. The calculation of CTT in R studio we use ltm package to calculate Cronbach’s coefficient, bruceR package for exploratory factor analysis, degree of variability, correlation coefficients are done using the appropriate formulas.

(1)Cronbach’s coefficient method: to analyze the items from the perspective of internal consistency, calculate the Cronbach’s coefficient α1 for each domain, and then compare it with the α2 coefficient of the domain after deleting this item, if α1 ≥ α2, evaluating it as a good item. If the subscale Cronbach’s α coefficient is above 0.7, it means that the scale has good reliability, between 0.6 and 0.7 means that the scale is acceptable, and if the α reliability coefficient is lower than 0.6, then consider modifying the scale.

(2) Degree of variability method: to analyze the items from a sensitivity perspective, calculate the standard deviation of each item, and evaluate those with a large degree of dispersion (> 0.90) as good items.

(3)Correlation coefficient method: In order to evaluate the independence or representativeness of the analyzed items, the correlation coefficients of the individual items with the scale scores were calculated. If the correlation coefficients of the items in the scale with the scores of the domains to which they belonged and with the total scale were > 0.5, it means that the correlation of the items with the domains to which they belonged and with the total scale was high, and this item could be rated as a good item.

(4) Exploratory factor analysis: In order to evaluate the representativeness of the analyzed items, according to the principle eigenvalue > 1, principal component analysis is used, and after orthogonal rotation with maximum variance, the factor loadings of each item are calculated. An item with a factor loading > 0.5 is considered a good item, and if the factor loading of an item in the scale is < 0.5, it means that the item does not have much influence on the latent variable to be measured. By exploratory factor analysis (EFA) of the minimum residual decomposition to test the unidimensionality of the scale. It is generally accepted that the unidimensionality assumption is largely met when the first factor explains more than 20–40% of the variance and the ratio of the first to second eigenvalue is greater than three [22].

### Item response theory(IRT)

Unlike the CTT, the IRT directly simulates the response of an item to its corresponding underlying trait, overcoming the shortcoming that CTT parameter estimation should depend on samples. Compared to the CTT, it can accurately estimate the measurement error of each item and each participant [18].

QLICD-AD (V2.0) is divided into four domains: physical functioning domain, psychological functioning domain, social functioning domain, and the specific module, and each item is scored using a five-point Likert scale, which is in line with the characteristics of the ordered multiclassification, and in this study, we can use the GRM rank-response model of the hierarchical multiclassification in the IRT [23]. The formula of the rating response model [24] as below:

$$ P\left({v}_{i}=k|\theta =t\right)=\frac{1}{1+\text{e}\text{x}\text{p}[-1.7{a}_{i}\left(t-{b}_{i,k}\right)]}-\frac{1}{1+\text{e}\text{x}\text{p}[-1.7{a}_{i}\left(t-{b}_{i,k+1}\right)]}$$

The hierarchical response model treats each item as a series of dichotomies (one minus the number of categories) and estimates each dichotomous 2-parameter model for each dichotom, corresponding to the lowest and highest categories, $$ P\left({v}_{i}=k|\theta \right)=0 $$and 1. $$ v$$ responses to multilevel scoring items 𝑖, $$ k$$ indicates a response option, $$ \theta $$(theta) is the latent variable measured by the item, a is the discriminant parameter, and b is the threshold parameter.

The amount of information, the average amount of information, the difficulty coefficient, and the degree of differentiation at different positions of each item were calculated to analyze the micro-evaluation of the items on the scale. We also estimated the TIF and the associated standard error of measurement (SE), which indicates the precision of the entire scale [25], to determine the level at which the QLICD-AD (V2.0) provided the most information. The parameters were estimated using the Marginal Maximum Likelihood Estimation (MMLE) method and the Expectation Maximization Algorithm (EM) [26].The computation and plotting of the IRT was done in R Studio in the mirt package, purrr package.

(1) The information amount of the items: reflects the amount of information that each item can provide in estimating the respondent’s ability, the larger the information amount, the smaller the standard error of measurement. In this paper, five points with values of -2, -1, 0, 1, and 2 are selected, and the values of the information function parameter $\theta$and its average value at these five points are calculated. Scale measurement information amount > 25 indicates that the quality of the measurement is good, information amount 16–25 indicates that the measurement is acceptable and information amount < 16 indicates that the measurement are poor [14, 19]. The QLICD-AD (V2.0) scale has a total of 40 items, and the average information amount of each item can be obtained by dividing 16 and 25 by 40, so that items with an average information amount > 0.63 (25/40) are judged to be excellent; <0.40 (16/40) are judged to be poor. However, we believe that this criterion is too strict. In this study, the total information amount of the scale was considered to be 5 based on a reliability equal to 0.8, and the average information amount of each item was 0.125 (5/40). Accordingly, when the mean information amount of an item was greater than 0.125, the item was evaluated as “good” and those less than 0.125 (5/40) were evaluated as “poor”.

(2) Difficulty coefficient b: the scale adopts a five-point equidistant scoring method, and each item has four difficulty coefficients, which are b1, b2, b3 and b4, with the increase of difficulty level (b1→b4), the difficulty coefficients corresponding to each item should show a monotonically increasing trend, and the items with the range of [-4, 4] are good; Degree of differentiation a: The greater the degree of differentiation, the greater the amount of information of the cued items, and the items with a degree of differentiation > 0. 5 are considered good.

(3) Item Characteristic Curve(ICC): It is used to describe the functional relationship between a subject’s latent traits and the probability of response. The Item Information Curve (IIC) describes the fact that a larger area under the curve indicates a higher degree of measurement accuracy. Test Information Function (TIF) reflects the precision of the test at various levels for the trait being measured. In general, the quality of the scale was considered high when the total information was 25 or more, and the quality of the scale was considered acceptable when the total information was between 16 and 25 [27, 28]. In addition, a list of conversions between raw total scores and IRT trait scores was calculated using the Expected A Posteriori (EAP) method of Bayesian estimation [20]. The IRT scores were calculated by integrating the parameter estimates (a, b, c) for each item, which means that the corresponding IRT scores are an interval of the same total score.

## Results

### Patient’s characteristics

A total of 120 AD patients with anxiety disorders aged 15–63 years agreed to participate in the study. Among the studied patients, 74 (61.7%) were males and 46 (38.3%) females; 30% were unmarried, and the divorced and widowed were 1. 7 and 2.5%, respectively; family economy was predominantly middle class, totaling 67 (55.8%); occupation was half occupied by farmers and laborers, 30 (25.0%) and 29 (24.2%), respectively, and the total detection rate of complete anxiety symptoms was 61.7%. See Table 2 in detail.

**Table 2:** Socio-demographic characteristics of the participants (*N* = 120)

Items	*n*(%)	Items	*n*(%)
**Gender**		**Job**	
Male	74(61.7)	Worker	29(24.2)
Female	46(38.3)	Peasant	30(25.0)
**Age**		Teacher	7(5.8)
15 ∼ 29	41(34.2)	Official	6(5.0)
30 ∼ 44	50(41.7)	Freelance	10(8.3)
≥ 45	29(24.1)	Student	17(14.2)
**Education**		Other	21(17.5)
Primary school	15(12.5)	**Medical insurance**	
Middle school	45(37.5)	Self-provided	11(9.2)
High school	32(26.7)	Urban worker medical insurance	33(27.5)
2 year college	10(8.3)	Commercial health insurance	2(1.6)
Undergraduate and above	18(15.0)	Rural cooperative medical insurance	74(61.7)
**Marriage**		**Diagnosis**	
Unmarried	36(30.0)	Anxiety disorder	74(61.7)
Married	79(65.8)	Anxiety disorder + Gastritis	15(12.5)
Divorced	2(1.7)	Anxiety disorder + Other	31(25.8)
Widowhood	3(2.5)	**treatments**	
**Economic status**		Anxiolytic	5(4.2)
Poor	43(35.9)	Anxiolytic + Complementary treatment	9(7.5)
Middle	67(55.8)	Anxiolytic + Improve sleep	54(45)
Good	10(8.3)	Anxiolytic + Complementary treatment + Improve sleep	45(37.5)
		Complementary treatment	7(5.8)

### Scores of the QLICD-AD (V2.0)

**Fig. 1:**
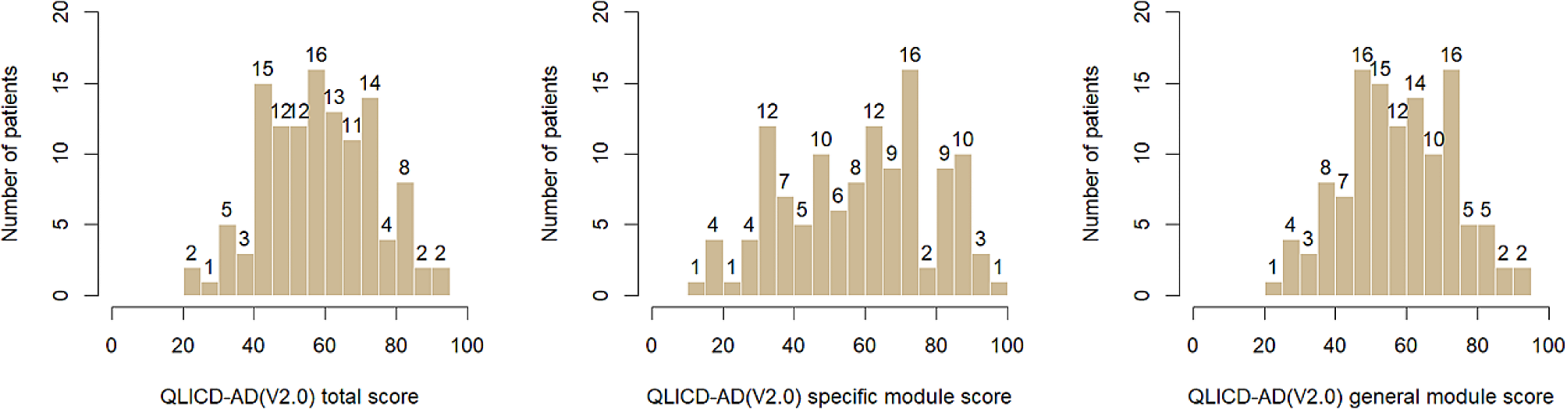
Histogram of total and module scores of the QLICD-AD (V2.0)

The overall mean score of the QLICD-AD (V2.0) was 58.44 ± 15.06 with a range of 24.47 to 91.49; a mean score of the general module was 57.52 ± 15.24 with a range of 20.31 to 90.63; and a mean score of the specific module was 58.58 ± 20.69 with a range of 12.50 to 95.83. General module skewness:-0.564 < 0;kurtosis:0.232 > 0. skewness z-score:2.058;kurtosis z-score:0.429,negative skewness, peak; specific module skewness:-0.567 < 0;kurtosis:0.241 < 0. skewness z-score:2.069;kurtosis z-score:0.445,negative skewness, flat peak; general module skewness:-0. 241 < 0. kurtosis:-0.241 < 0. skewness z-score:2.069; kurtosis z-score:0.445,negative skewness, flat peak; skewness of the whole QLICD-AD (V2.0):-0.602 < 0; kurtosis:0.194 > 0. skewness z-score: 2.197; kurtosis z-score: 0.359, negative skewness, sharp peak. There was no “floor effect” or “ceiling effect” in the overall score or in the scores of each domain/module. See Fig. 1 in detail.

### Classical test theory analyses

Based on the results of CTT analysis, Cronbach’s coefficient alpha value of QLICD-AD (V2.0) scale is 0.931. The physical functioning neighborhood Cronbach’s coefficient alpha value is 0.706, and Cronbach’s coefficient alpha coefficient of the 9 items after the deletion of a certain item ranged from 0.655 to 0.692; psychological functioning neighborhood Cronbach’s coefficient alpha value is 0.855, the Cronbach’s coefficient alpha after the deletion of an item in 11 items ranged from 0. 825 to 0.866, and GPS3 and GPS10 were not satisfied; the Cronbach’s coefficient alpha value for social functioning neighborhood was 0.758, the Cronbach’s alpha coefficient after deleting an item in 8 items ranged from 0.699 to 0.774, and GSO6 was not satisfied; and the Cronbach’s coefficient alpha value for the specific module neighborhood was 0.865, and the Cronbach’s alpha coefficient of 12 items after deleting one item ranged from 0.847 to 0.863.

40 items satisfied the degree of variability method. The correlation coefficients between the items and the scores on the total scale ranged from 0.321 to 0.711, with 10 items < 0.5 and the other 30 items > 0.5, which is a good result. The factor analysis showed that the KMO value = 0.804, Barlett’s spherical test$$ {x}^{2}$$ = 2618.627,$$ P $$< 0.001, and 30 items satisfied the factor analysis.

**Table 3:** Results of QLICD-AD (V2.0) items analysis based on four methods under CTT

Items	Variability	Correlation coefficient	Factor Analysis	Cronbach’s α coefficient		Item evaluation
Physical Function				0.706		
GPH1	1.071*	0.482	0.382	0.692*	II	
GPH2	1.073*	0.442	0.484	0.691*	II	
GPH3	1.328*	0.551*	0.541*	0.674*	I	
GPH4	0.984*	0.479	0.485	0.691*	II	
GPH5	1.229*	0.559*	0.590*	0.676*	I	
GPH6	0.970*	0.481	0.578*	0.691*	I	
GPH7	1.388*	0.648*	0.573*	0.655*	I	
GPH8	1.076*	0.582*	0.496	0.676*	I	
GPH9	1.221*	0.551*	0.608*	0.680*	I	
Psychological Function				0.855		
GPS1	1.238*	0.491	0.565*	0.853*	I	
GPS2	1.309*	0.604*	0.568*	0.844*	I	
GPS3	1.181*	0.321	0.562*	0.866	II	
GPS4	1.235*	0.711*	0.698*	0.835*	I	
GPS5	1.325*	0.613*	0.627*	0.844*	I	
GPS6	1.391*	0.703*	0.699*	0.836*	I	
GPS7	1.327*	0.822*	0.735*	0.825*	I	
GPS8	1.374*	0.786*	0.709*	0.828*	I	
GPS9	1.401*	0.700*	0.693*	0.837*	I	
GPS10	1.299*	0.457	0.529*	0.856	II	
GPS11	1.192*	0.697*	0.658*	0.835*	I	
Social Function				0.758		
GSO1	1.322*	0.556*	0.636*	0.729*	I	
GSO2	1.014*	0.681*	0.471	0.738*	I	
GSO3	0.928*	0.553*	0.476	0.742*	I	
GSO4	1.053*	0.445	0.473	0.713*	II	
GSO5	1.214*	0.706*	0.543*	0.699*	I	
GSO6	1.382*	0.481	0.446	0.774	III	
GSO7	1.398*	0.534*	0.568*	0.753*	I	
GSO8	1.170*	0.836*	0.569*	0.709*	I	
Specific module				0.865		
AD1	1.343*	0.632*	0.546*	0.856*	I	
AD2	1.267*	0.541*	0.464	0.861*	I	
AD3	1.405*	0.685*	0.583*	0.850*	I	
AD4	1.291*	0.660*	0.713*	0.853*	I	
AD5	1.172*	0.533*	0.380	0.861*	I	
AD6	1.281*	0.727*	0.644*	0.847*	I	
AD7	1.292*	0.715*	0.630*	0.849*	I	
AD8	1.281*	0.588*	0.540*	0.854*	I	
AD9	1.300*	0.589*	0.533*	0.857*	I	
AD10	1.466*	0.725*	0.644*	0.849*	I	
AD11	1.327*	0.592*	0.473	0.858*	I	
AD12	1.192*	0.491	0.530*	0.863*	I	

* selected by this method. Item evaluation: I=excellent, II=good, III=poor

In summary, since GSO6 satisfies only one statistical method, further major change was needed. GPH1,GPH2,GPH4,GPS3,GPS10,GSO4 satisfy both statistical methods, need to make appropriate adjustments. There were a total of 33 items that satisfied at least 3 statistical methods. See Table 3 in detail.

**Fig. 2:**
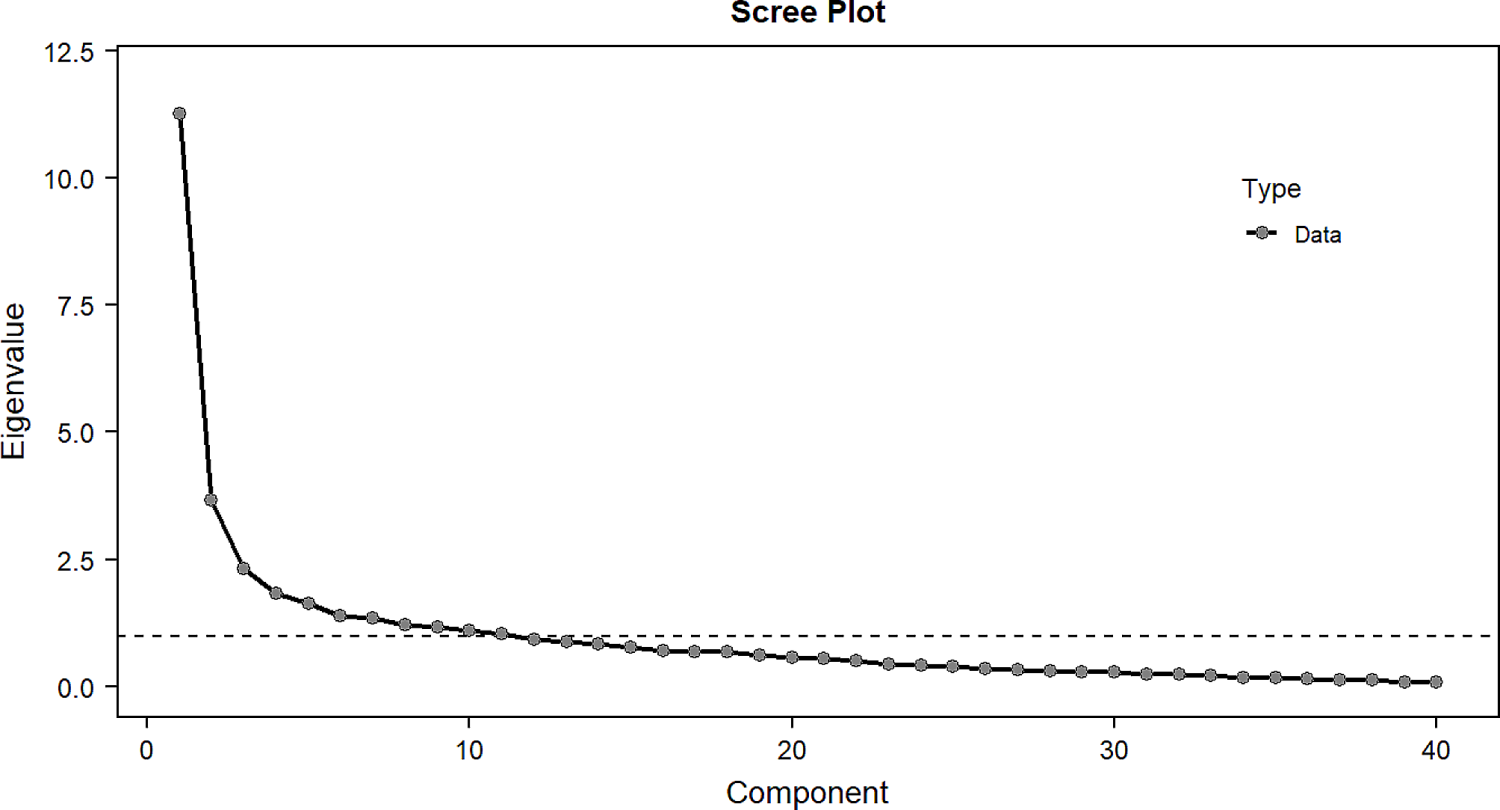
Scree plot of QLICD-AD (V2.0)

The results of unidimensionality test in this study showed that the ratio of the first and second Eigenvalue > 3. See Fig. 2 in detail.

### IRT analyses

In this study, the GRM of IRT was used to calculate the differentiation, difficulty coefficient and average information amount of each item.

### Discrimination and difficulty

As can be seen in Table 4 in detail, the differentiation of the 40 items ranged from 0.35 to 1.94, with 38 items having a differentiation > 0.50 and 2 items (GPH6 and GPS3) having a lower differentiation. The difficulty of each item ranged from − 12.134 to 5.072, and there were 32 items that met the − 4 to 4 and monotonically increasing trend, while GPH6, GPH8, GPS3, GSO2-GSO4, AD2, and AD5 did not meet the requirements.

**Table 4:** Estimates of discrimination and difficulty parameters of QLICD-AD(V2.0) based on IRT GRM

Items	a	b1	b2	b3	b4	Items	a	b1	b2	b3	b4
GPH1	0.769*	-3.227	-1.074	1.412	3.451	GSO1	0.670*	-3.853	-1.644	0.337	1.326
GPH2	0.897*	-1.381	0.382	2.477	3.752	GSO2	0.610	-6.475	-4.770	-1.177	0.972
GPH3	1.00*	-2.631	-1.426	-0.542	0.996	GS03	0.725	-6.184	-4.852	-0.486	1.461
GPH4	0.673*	-3.132	-1.868	1.380	3.403	GSO4	0.823	-5.08	-3.284	-1.874	-0.575
GPH5	1.378*	-1.544	-0.419	0.401	2.490	GSO5	1.111*	-3.699	-1.808	-0.761	0.264
GPH6	0.407	-12.134	-6.842	-3.986	-3.100	GSO6	0.858*	-1.909	-1.132	-0.126	2.273
GPH7	0.651*	-3.681	-2.411	-1.451	0.132	GSO7	1.320*	-1.453	-0.793	-0.009	1.540
GPH8	0.795	-5.672	-3.129	-2.312	-1.890	GSO8	1.204*	-3.323	-2.161	-0.918	0.058
GPH9	1.266*	-1.453	-0.119	0.650	3.094	AD1	1.035*	-2.433	-1.029	-0.245	1.402
GPS1	0.773*	-3.301	-1.421	0.139	2.076	AD2	0.694	-4.025	-2.471	-0.966	0.789
GPS2	1.261*	-1.730	-0.647	-0.021	2.007	AD3	1.304*	-1.847	-0.984	-0.533	0.958
GPS3	0.346	-6.571	-2.215	2.356	5.072	AD4	1.927*	-1.694	-0.882	-0.077	1.150
GPS4	1.665*	-1.250	-0.251	0.549	2.376	AD5	0.648	-4.103	-2.353	-0.301	2.419
GPS5	1.541*	-1.959	-0.899	-0.354	1.015	AD6	1.487*	-1.342	-0.395	0.264	2.523
GPS6	1.893*	-0.845	-0.246	0.253	1.792	AD7	1.451*	-1.519	-0.204	0.223	2.202
GPS7	1.923*	-1.410	-0.442	0.026	1.630	AD8	0.972*	-2.644	-1.646	-0.816	1.216
GPS8	1.939*	-1.566	-0.741	-0.152	0.978	AD9	1.063*	-2.652	-1.606	-0.687	0.919
GPS9	1.990*	-0.987	-0.183	0.274	1.486	AD10	1.460*	-1.375	-0.678	-0.033	1.109
GPS10	0.875*	-3.018	-1.013	0.329	1.511	AD11	0.716*	-3.494	-1.724	-0.336	1.347
GPS11	1.684*	-2.075	-1.172	-0.366	1.278	AD12	1.130*	-2.903	-1.755	-0.729	0.912

a: Degree of differentiation; b1, b2, b3 and b4 difficulty level. * selected item

### Average information amount

In this study, 35 out of 40 items had mean information amount > 0.125, 11 of them were judged as excellent, 24 were judged as fair and the remaining 5 (GPH1,GPH6,GPH8,GPS3,GSO2) were judged as poor. See Table 5 in detail.

**Table 5:** Information amount at different points($\varvec{\theta }$) of items of the QLICD-AD (V2.0)

Item	$\varvec{\theta }$					Average information	Item evaluation
	-2	-1	0	1	2		
GPH1	0.104	0.115	0.122	0.124	0.119	0.117	III
GPH2	0.130	0.194	0.261	0.319	0.345	0.250	II
GPH3	0.352	0.522	0.437	0.247	0.132	0.338	II
GPH4	0.113	0.121	0.131	0.140	0.139	0.129	II
GPH5	0.275	0.582	0.754	0.499	0.299	0.482	I
GPH6	0.034	0.027	0.021	0.016	0.012	0.022	III
GPH7	0.181	0.180	0.138	0.090	0.054	0.129	II
GPH8	0.225	0.171	0.099	0.050	0.025	0.114	III
GPH9	0.220	0.461	0.655	0.464	0.252	0.410	I
GPS1	0.183	0.224	0.226	0.189	0.138	0.192	II
GPS2	0.284	0.579	0.632	0.369	0.212	0.415	I
GPS3	0.030	0.032	0.033	0.033	0.032	0.032	III
GPS4	0.266	0.730	1.225	0.770	0.449	0.688	I
GPS5	0.400	0.862	0.856	0.425	0.211	0.551	I
GPS6	0.173	0.760	1.754	0.785	0.430	0.780	I
GPS7	0.331	1.109	1.897	0.736	0.475	0.910	I
GPS8	0.396	1.252	1.578	0.701	0.301	0.846	I
GPS9	0.218	0.749	1.716	0.905	0.414	0.801	I
GPS10	0.158	0.211	0.240	0.214	0.153	0.195	II
GPS11	0.648	1.408	1.000	0.547	0.341	0.789	I
GSO1	0.129	0.156	0.165	0.146	0.109	0.141	II
GSO2	0.157	0.141	0.121	0.098	0.074	0.118	III
GS03	0.148	0.139	0.138	0.129	0.106	0.132	II
GSO4	0.323	0.281	0.179	0.097	0.051	0.186	II
GSO5	0.373	0.501	0.417	0.225	0.104	0.324	II
GSO6	0.207	0.301	0.293	0.204	0.128	0.227	II
GSO7	0.271	0.611	0.680	0.391	0.211	0.433	II
GSO8	0.622	0.754	0.501	0.227	0.094	0.439	II
AD1	0.281	0.454	0.458	0.291	0.159	0.329	II
AD2	0.221	0.227	0.181	0.122	0.077	0.165	II
AD3	0.351	0.752	0.666	0.308	0.150	0.445	II
AD4	0.503	1.426	1.464	0.786	0.369	0.910	I
AD5	0.198	0.205	0.176	0.136	0.101	0.163	II
AD6	0.254	0.687	0.939	0.478	0.258	0.523	II
AD7	0.250	0.579	0.910	0.555	0.284	0.516	II
AD8	0.427	0.602	0.421	0.222	0.129	0.360	II
AD9	0.394	0.559	0.434	0.238	0.128	0.350	II
AD10	0.270	0.779	1.025	0.504	0.217	0.559	II
AD11	0.203	0.247	0.231	0.169	0.108	0.192	II
AD12	0.492	0.604	0.425	0.241	0.137	0.380	II

Item evaluation: I=excellent, II=good, III=poor

### Item characteristic/ information curve

**Fig. 3:**
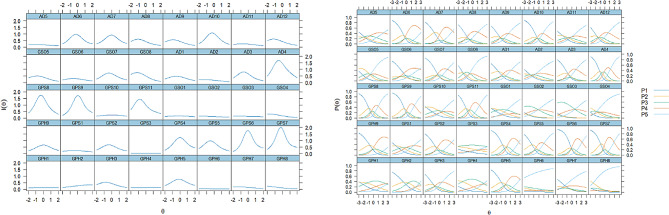
Item information curve (IIC) and item characteristic curve (ICC) of QLICD-AD (V2.0)

Item Characteristic Curve(ICC) Expresses the probability of each option being selected as a contribution to the estimated basis function. Figure 3 shows the ICC and the Item Information Curve(IIC) for all items. The smallest area under the curve shown on the left is for items of GPH1,GPH6,GPH8,GPS3,GSO1-GSO3,AD2,AD5, indicating measurement accuracy is low. Figures P1-P5 on the right show different response options GPS3,GPS8,GPH6,GPH8,GSO4,AD2 Response probabilities are similar across categories and a response always has the highest probability at higher levels of the continuum.

### Test information function

**Fig. 4:**
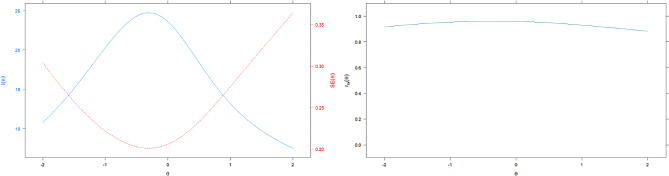
Test information function (TIF) and reliability of QLICD-AD (V2.0)

Figure 4 shows the test information function and measurement error. It can be seen that information is highest (standard error lowest) in the range of -1 to 0 on the z-score metric, all marginal reliabilities for this scale were > 0.8.

## Discussions

For many years, CTT and IRT have been the two major methods used for test and scale construction and development in the educational, behavioral and social sciences [29]. CTT and IRT are also the two most classical theories in the field of scale development and are commonly used for item analysis and screening. CTT evaluates scale from a macro perspective [30]. It is accurate enough in most cases, but theoretical hypothesis is weak and error index is general and single. The biggest disadvantage of it is that it has large dependence on samples, IRT overcomes it. IRT calculates the discrimination, difficulty and information of each item from the micro level. The item parameter estimation is independent of the sample, which can accurately estimate the measurement error of each item and test for each subject, evaluate item more accurately. QLICD-AD(V2.0) items have five degrees, IRT could perform a more accurate analysis and estimation of the non-linear model and better meet the needs of modern analysis [31]. CTT and IRT complement each other, and the combination of the two can better assess items.

From the results of CTT analysis, seven items (GPH1, GPH2, GPH4, GPS3, GPS10, GSO4, GSO6) did not satisfy the three statistical methods in CTT. The correlation coefficients of the items GPH1,GPH2,GPH4,GPS3,GPS10,GSO4,GSO6 are small, and the representativeness and independence of the items are poor. The factor loadings of GPH1,GPH2,GPH4,GSO4,GSO6 are small, and the representativeness of the items is poor. There is a role for reducing the internal consistency of the dimension for GPS3,GPS10,GSO6. For GSO6, in one study, more than half of those who remained disordered at follow-up had significant health care costs, treatment-resistant symptoms, and severely impaired quality of life [32]. However, considering that the four statistical methods satisfy at least three of the items rated as good quality, the final CTT method determines that seven items are subject to further optimization.

From the results of IRT analysis, the average amount of information of GPH1, GPH6, GPH8, GPS3, GSO2 was too low. The difficulty or differentiation of items of GPH6, GPS3 did not meet the judging criteria. The difficulty coefficients of GPH6, GPH8, GPS3, GSO2-GSO4, AD2, AD5 were not within the range of the judging criteria. Together with the IIC non-compliant graph items of GPH1, GPH6, GPH8, GPS3, GSO1-GSO3, AD2, AD5 and the ICC non-compliant graph items of GPH8, GPS3, GPS8, GSO4, AD2, the differentiation of the 38 items meets the judging criteria and the items provide a greater amount of information.

**Table 6:** CTT and IRT unlisted items summary

Items	CTT				IRT			Total
	Variability	Correlation coefficient	EFA	Cronbach’s α	Discrimination	difficulty coefficient	Average information	
GPH1	✓	X	X	✓	✓	✓	X	E
GPH2	✓	X	X	✓	✓	✓	✓	E
GPH4	✓	X	X	✓	✓	✓	✓	E
GPH6	✓	X	✓	✓	X	X	X	D
GPH8	✓	✓	X	✓	✓	X	X	E
GPS3	✓	X	✓	X	X	X	X	D
GPS10	✓	X	✓	X	✓	✓	✓	E
GSO2	✓	✓	X	✓	✓	X	X	E
GSO3	✓	✓	X	✓	✓	X	✓	E
GSO4	✓	X	X	✓	✓	X	✓	E
GSO6	✓	X	X	X	✓	✓	✓	E
AD2	✓	✓	X	✓	✓	X	✓	E
AD5	✓	✓	X	✓	✓	X	✓	E

✓:Status of Selection; X: Non-selection; D = Delete; E = Enhancement

In summary(Table 6), combining the results of CTT and IRT analyses, among QLICD-AD (V2.0) 40 items, there are 32 items with good performance, 6 items (GPH1, GPH8, GSO2, GSO4, AD2, AD5) need to be further optimized, item GPH6,GPS3 should be deleted due to the number of tests do not meet the requirements. The remaining items are of better quality. Although the results showed that the QLICD-AD (V2.0) could be effectively used to measure patients with anxiety disorders, for the items that needed to be modified and deleted, the anxiety disorder experts in the group discussed the statistical results and suggested modifications to avoid errors caused by relying solely on statistical analysis and to improve the representativeness and reliability of the items.

This study has used two theories to evaluate items of the QLICD-AD(V2.0) for relatively comprehensive and complementary, but the sample size and scope of the collection are still limited. Sample size for IRT analysis of items generally requires 250 cases [33]. Due to time, manpower and other reasons, this research does not meet the requirements of a large sample size. In order to make the scale evaluation more accurate and reliable, the sample size can be increased for further analysis and evaluation. In addition, the subjects in this study were only selected from hospital inpatients. Further large-scale research is needed for other settings and populations, such as outpatients in hospitals or local clinics. The next step is to adjust the QLICD-AD(V2.0) based on the above results. In the future, we will work with psychiatric departments of hospitals in different provinces of China and local communities to expand the population coverage, so that the QLICD-AD(V2.0) can become a suitable scale for measuring anxiety disorders in China.

## Data Availability

No datasets were generated or analysed during the current study.

## Abbreviations

AD: Anxiety disorder
QOL: Quality of life
CTT: Classical test theory
IRT: Item response theory
GRM: Graded response model
EFA: Exploratory factor analysis
ICC: Item Characteristic Curve
IIC: Item Information Curve
TIF: Test Information Function
EAP: Expected A Posteriori
